# Effect of the Preparation Conditions on the Catalytic Properties of CoPt for Highly Efficient 4-Nitrophenol Reduction

**DOI:** 10.3390/ma15186250

**Published:** 2022-09-08

**Authors:** Oana-Georgiana Dragos-Pinzaru, Gabriela Buema, Daniel Gherca, Ibro Tabakovic, Nicoleta Lupu

**Affiliations:** 1 National Institute of R&D for Technical Physics, 700050 Iasi, Romania; 2 ECE Department, University of Minnesota, Minneapolis, MN 55435, USA

**Keywords:** CoPt thin films, electrodeposition, catalysts, catalytic activity, 4-NP reduction

## Abstract

CoPt alloys with Pt contents from 15 to 90% were prepared using low-cost electrochemical deposition. Different samples were synthesized from electrochemical baths at pH = 2.5 and 5.5 in a solution with and without saccharin as an additive. The morphology, composition and crystalline structure of the as-prepared samples were investigated by High Resolution—Scanning Electron Microscopy (HR-SEM), Atomic Force Microscopy (AFM), Ultra-high Resolution—Transmission Electron Microscopy (UHR-TEM), Energy-Dispersive X-ray Spectroscopy (EDX), and X-ray Diffraction (XRD). XRD investigations revealed that fcc crystalline structure transforms into hcp crystalline structure when the pH of the electrochemical bath is increased from 2.5 to 5.5 as well as when saccharin is added to the electrochemical bath. The catalytic performance of the CoPt alloys for the nitro to amino phenol compounds conversion was investigated for all the prepared samples, and the results show that the conversion degree increases (from 11.4 to 96.5%) even though the Pt content in the samples decreases. From the samples prepared from the electrochemical bath with saccharin, a study regarding the effect of contact time was performed. The results indicated that after only 5 min, the CoPt sample prepared at pH = 5.5 in the presence of saccharin completely converted the nitro compound to an amino compound.

## 1. Introduction

Metallic materials in the form of thin films, nanoparticles, nanowires, nanorods, or nanotubes are employed in a variety of applications in catalysis, nanomedicine or electronics. The physical characteristics of nanomaterials differ from those of the same materials in bulk or single crystals and are heavily influenced by their size and geometry [1,2,3,4,5]. The change in the properties is due to the increase in the surface-to-volume ratio, the special shape of the nanostructured materials, the aspect ratio of the nanostructure or the synthesis conditions [6,7,8].

The CoPt alloys are one of the most studied materials due to their possible applications in BPM (Bit Patterned Media) for high-density magnetic recording [9,10], microelectromechanical systems (MEMS) for sensing and actuating devices [9,11], Magnetic Resonance Imaging (MRI) contrast agents [1,12], or as catalysts [13,14,15,16].

In the field of catalysis, the CoPt alloy has been widely studied in the last years for possible application as a catalyst for a Fisher–Tropsch reaction [17], imines synthesis [18], carbon nanotubes and nanofibers preparation [19,20], Li-O_2_ batteries [21], hydrogen generation [22,23,24,25], Oxygen Reduction Reaction [26,27], Methanol Oxidation Reaction [28,29,30,31] as well as for the catalytic reduction of 4-Nitrophenol [15,32]. The most used synthesis route for CoPt alloy preparation is chemical reductions [33] and electrochemical deposition [11,31,34,35,36]. Electrochemical synthesis represents a good route to preparing metallic alloys [37,38] because it is easily applied, does not require a high vacuum and the fact that the growth speed and the chemical composition of the alloys can be controlled.

Water pollution with organic compounds continuously increases with the discharge of large quantities of chemical, pharmaceutical or agrochemical products, which comprise a significant part of the total organic pollutants from industry. Organic pollutants, such as surfactants, heterocyclic, phenolic or aromatic nitro-compounds, are dangerous to humans or ecosystems and difficult to degrade by microorganisms. The nitroaromatic compounds are important intermediates for several industries and are used to prepare fungicides, pesticides, dyes and drugs.

**Scheme 1:**
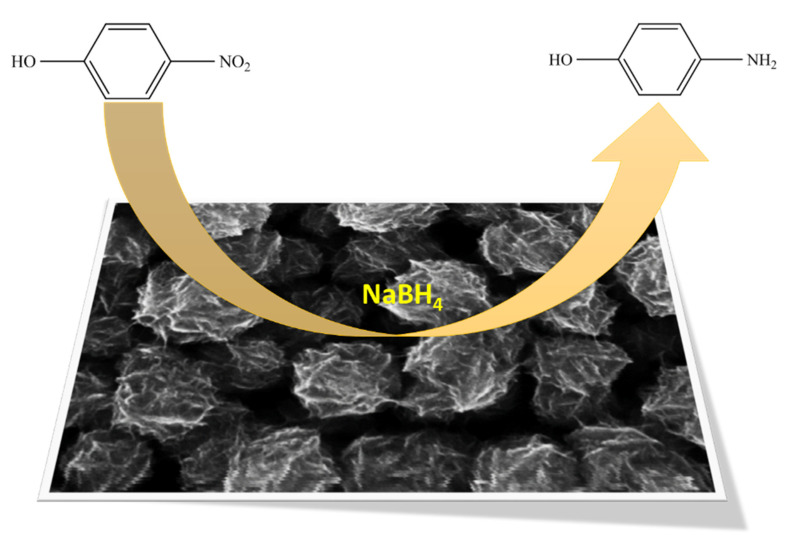
Schematic design of the 4-NP reduction with an aqueous solution of NaBH_4_, using the CoPt catalyst.

One of the most common aromatic nitro-compounds pollutants is 4-Nitrophenol (4-NP), which is widely used in the pharmaceutical and chemical industry as it is very carcinogenic and tends to persist in water and soil. In addition, 4-NP can cause headaches, drowsiness, nausea and cyanosis in the lips, ears, and fingernails [39]. One of the methods developed by researchers for 4-NP removal from contaminated waters is a catalytic reduction—a simple and fast approach that can be used for aromatic nitro-compounds removal, acting through the conversion of nitro-group to amino-group. In fact, the chemical reduction of 4-Nitrophenol (used to manufacture drugs—e.g., acetaminophen, fungicides, methyl and ethyl parathion insecticides, or dyes) has not only the advantage of depolluting the wastewaters, but the reaction product, 4-Aminaphenol (4-AP), is a useful compound working as an anticorrosion lubricant, intermediate for drugs or photographic developer. In this context, developing a new catalyzer able to reduce the 4-NP into 4-AP (a complete nontoxic compound) with better performance becomes necessary. At the same time, the 4-NP reduction by the NaBH_4_ reaction (presented in Scheme 1) is widely used by researchers as a test reaction to evaluate the catalytic properties of various catalysts [40,41,42,43].

According to the literature data, the conventional materials used as catalysts for nitro-compounds hydrogenation are noble metals in general and Pt-group metals in particular, in various forms such as wires [44], rods [45], prisms [46], plates [47], polyhedrons [48] or branched nanostructures [49]. Noble metals-based catalysts, such as platinum, are the most widely used in heterogeneous catalysis for their exceptional performance for H_2_ generation [50,51], but they are limited because of the huge price and low availability on the market. In this context, replacing platinum with other metals (especially non-noble metals) with comparable catalytic performances is a major challenge for the research community. The synthesis of new bimetallic catalysts by partially replacing the noble metal with a non-noble metal can be a reliable solution to solve the noble metals shortage; studies published in the literature show that the Pt can be successfully replaced by Cu [52], Ni [53], Fe [54] or Co [55]. However, the catalytic properties of bimetallic catalysts strongly depend on the alloy composition and arrangement of the two metal atoms on the particle’s surface; consequently, they depend on the crystalline structure of the alloy [56].

Our work aims to prepare CoPt thin films with a low content of Pt but with optimum catalytic properties for the chemical reduction of 4-Nitrophenol.

In this work, we will show that the CoPt alloy composition and crystalline structure can be tuned by varying the electrodeposition parameters, such as electrodeposition potential and electrochemical bath composition. The objective is to find the appropriate preparation conditions that lead to synthesizing catalysts with optimum properties. More specifically, we have prepared CoPt thin films using electrodeposition from an electrochemical bath with different characteristics (such as pH value or presence of additives). The saccharin is an additive known as a smoothening and a stress relieving agent drastically influences the morphology, internal stress, hardness, microstructure and crystalline structure of the materials prepared when added into the electrochemical bath [57,58,59]. The choice of saccharin as an additive was made considering the previously presented literature to date, but also our team’s results, where it was demonstrated that the use of saccharin as additives during the electrodeposition of CoPt thin film leads to the preparation of hexagonal CoPt thin layers without cracks [31,36]. A complete morphological, compositional and structural characterization was conducted. Next, the catalytic performance of the as-prepared alloys for the 4-NP reduction was investigated. Remarkably, the catalytic performance of CoPt alloy increases despite the samples’ decrease in the Pt content. This behavior can be attributed to the change in the crystalline structure as well as to the differences in the surface roughness and crystallite size as a function of the electrochemical bath composition.

## 2. Materials and Methods

All reagents for synthesis and analysis were provided by Alfa Aesar and used without further purification. A stable aqueous hexachloroplatinate CoPt-solution at pH 5.5 containing 0.4 M H_3_BO_3_ (99.8%), 0.3 M NH_4_Cl (99.5%), 0.1 M CoSO_4_ 7H_2_O (98.0%), 0.00386 M H_2_PtCl_6_ (99.9%), with 0.00389 M sodium saccharin (99.0%), was prepared according to the recently published procedure [34]. The CoPt solution at pH 5.5 was prepared from the solution at pH 2.5 with the addition of 0.1 M NaOH (97.0%). The pulse electrodeposition was carried out by exposing the 1.0 cm^2^ sputtered Au seed layer on oxidized Si wafers with a 5 nm Ta adhesion layer to the quiescent plating solution. The pulse electrodeposition was performed in a 100 mL closed three-electrode cell with a platinum wire as a counter electrode, an Au seed layer as a working electrode, and a saturated calomel electrode (SCE) for reference. The applied potential was controlled with a Bipotentiostat/Galvanostat HEKA PG 340. The CoPt alloys were prepared from CoPt quiescent solution with and without saccharin additive.

Saccharin is used in order to facilitate the control of the crystalline structure of the CoPt alloy. The electrodeposition was carried out by using a pulsed potential of −0.8 V/SCE during 2.5 s time-on and the “rest” potential of −0.1 V/SCE during 1 s time-off. The time-off is necessary for a good “recovery” of the diffusion layer after the time-on. A KLA Tencor Alpha Step IQ profilometer was used to measure the as-deposited thin layers’ thicknesses. A HR-SEM (High Resolution–Scanning Electron Microscopy), NEON40EsB CrossBeam System from Carl Zeiss, was used to examine the microstructure of the as-prepared alloys. The Energy-Dispersive X-ray Spectroscopy (EDX) measurements inside the same HR-SEM machine were used to estimate the elemental composition. X-ray diffraction (XRD) was used to investigate the crystalline structure of the electrodeposited materials with a Bruker AXS D8-Advance X-Ray Diffractometer with parallel optical geometry and Cu K radiation (=1.5406), while the surface roughness was analyzed by Atomic Force Microscopy (AFM), using an AFM XE-100 from Park Systems. Ultra-high Resolution–Transmission Electron Microscopy was performed using the LIBRA^®^200MC system from Carl Zeiss.

The catalytic performance test of the 4-NP using NaHB_4_ (98.00%) was performed using a Perkin Elmer Lambda 35 UV/VIS spectrophotometer as follows: 2.5 mL of distilled water were put in contact with each material and mixed with 40 μL of 4-NP (10 mM) and 0.5 mL of NaHB_4_ (freshly aqueous solution of 0.2 M). The yellow mixture generated was intermittently stirred at room temperature for 120 min. The solution was analyzed using the UV–VIS method in the 300–700 nm range. The purity of the 4-Nitrophenol used during the experiments was 98.0%.

## 3. Results

### 3.1. Morphological and Compositional Characterization

**Scheme 2:**
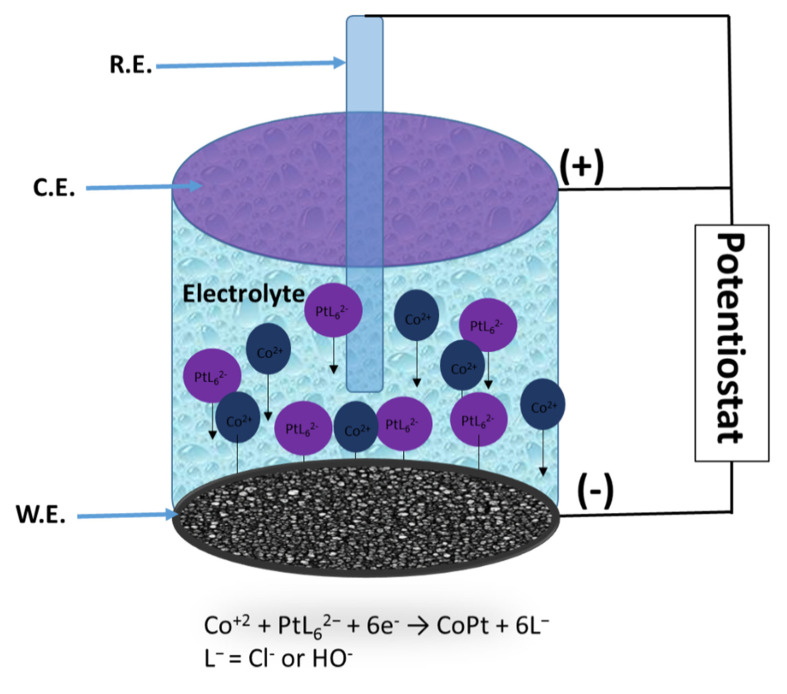
Schematic diagram of the CoPt thin films synthesis.

The alloy morphology, roughness, composition and thickness are important parameters for analysis since the catalytic properties of the materials are influenced by them. In this regard, very fine control of the synthesis parameters is necessary to obtain alloys with reproducible properties. The schematic diagram of the fabrication and formation of CoPt thin films is presented in Scheme 2. The electrodeposition is carried out in a three-electrode electrochemical cell consisting of a Pt wires as a counter electrode (C.E.), a Saturated Calomel Electrode—SCE as reference (R.E.) and an Au thin film as a working electrode (W.E.).

**Figure 1:**
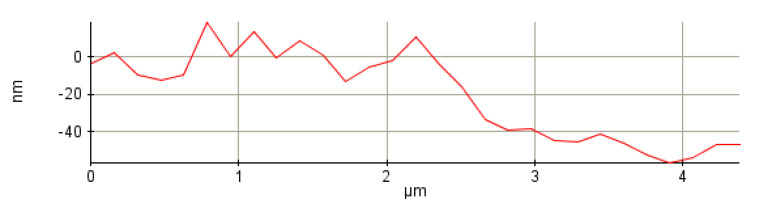
Line profile of 50 nm CoPt thin film prepared at pH = 5.5 in the presence of saccharin.

During this study, four different samples were prepared, with the catalytic activities of these samples being evaluated. All the samples were prepared at −0.8 V/SCE, with two of them from a solution at pH = 2.5 (one sample in the absence of saccharin–S1 and one in the presence of saccharin–S2) and the others from a solution at pH = 5.5 (one sample in the absence of saccharin–S3 and one in the presence of saccharin–S4). Since thin film properties are a function of its thickness, the CoPt thin layers with a thickness of 50 nm were investigated to compare the catalytic properties of materials prepared under various conditions. The synthesis time was calibrated for each electrochemical bath composition prior to electrodeposition. For the present study, we used CoPt electrodeposited thin layers having 50 nm thickness as measured by profilometry. Figure 1 presents the as-measured thickness profile of the CoPt thin layer prepared at pH = 5.5 in the presence of saccharin (the lines profiles for all the prepared samples are presented in the Supplementary Materials in Figure S1).

**Figure 2:**
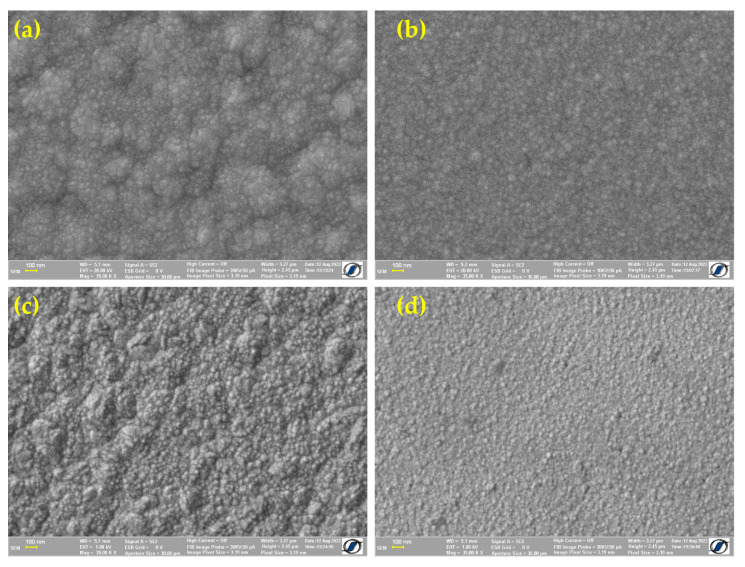
HR-SEM images of electrodeposited CoPt alloy for various electrodeposition solutions. (**a**) pH = 2.5 solutions without saccharin addition, (**b**) pH = 2.5 solutions with saccharin addition, (**c**) pH = 5.5 solutions without saccharin addition and (**d**) pH = 5.5 solutions with saccharin addition.

After electrodeposition, the CoPt electrodeposited surface was analyzed by HR-SEM and AFM, while EDX determined the composition. Figure 2 shows the HR-SEM images of the CoPt alloy electrodeposited from the electrochemical bath at pH = 2.5 without additive (Figure 2a), pH = 2.5 with additive (Figure 2b), pH = 5.5 without additive (Figure 2c) and pH = 5.5 with additive (Figure 2d), respectively.

The HR-SEM images clearly show that the electrodeposition bath composition influence the CoPt alloys’ thin film surfaces prepared at controlled potential (E = −0.8 V/SCE). As mentioned previously, the saccharin was used in order to reduce the stress, to prepare thin films without defects on the surface, as well as for good control of the crystalline structure of the as-deposited CoPt alloy. The SEM analysis of the samples prepared from the electrochemical bath without saccharin shows the presence of small cracks on the surface (Figure 2a,c). When the saccharin was added to the electrochemical bath, the surfaces of the samples were free of cracks.

**Figure 3:**
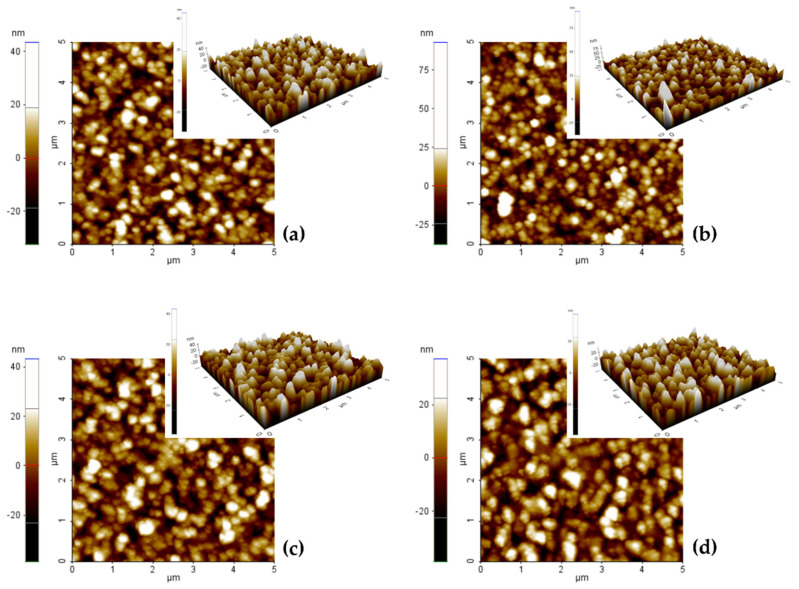
AFM images of electrodeposited CoPt alloy for various electrodeposition solution: (**a**) pH = 2.5 solutions without saccharin addition, (**b**) pH = 2.5 solutions with saccharin addition, (**c**) pH = 5.5 solutions without saccharin addition and (**d**) pH = 5.5 solutions with saccharin addition.

AFM images (presented in Figure 3) were used to study the root mean square (RMS) roughness of thin films. The most generally reported measurement of surface roughness is the root mean square roughness, which is defined as the standard deviation of the surface height profile from the average height [60].

We determined that the above-mentioned thin films have extremely good homogeneity. Based on the SEM and AFM surface analysis, we can conclude that the sample’s surface is completely covered with the CoPt alloy film. The surface roughness and the grain size (summarized in Table 1) are strongly influenced by the preparation conditions and less by the roughness of the substrate (Ra = 1.09 nm; data presented in Supplementary Materials, Figure S2). Thus, the roughness of the alloys prepared at pH = 2.5 (sample S1) from the solutions without the saccharin addition is 7.5 nm and slightly increases to 9 nm when saccharin is added to the electrochemical bath (sample S2), while the average grains size decrease from 55 to 50 nm.

The same results (a slight increase of the surface roughness) are obtained by the increase of the electrochemical bath pH from 2.5 to 5.5, the samples prepared in this synthesis conditions presenting a surface roughness of 9.2 nm (sample S3). The average grain size of the sample prepared at pH = 5.5 in the absence of saccharin is 90 nm. The samples prepared at pH = 5.5 and in the presence of saccharin (sample S4) present the highest roughness value, respectively, 10.6 nm, while the average grain size is 75 nm. Table 1 summarizes the physical and chemical properties of the as-prepared samples. It is clear that the sample’s roughness and average grain size are strongly correlated with the electrodeposition conditions and, respectively, with the alloy composition. Thus, the roughness value increase with the increase of the Co content of the sample. A similar evolution of the surface roughness as a function of the alloy composition for CoNi electrodeposited thin films was reported previously by Tebbakn et al. [61].

The compositional analysis of the catalysts was determined by EDX, with the obtained data being presented in Table 1. The EDX spectrum (presented in Supplementary Materials, Figure S3) of the as-prepared thin films indicates the presence of platinum and cobalt in different proportions of the preparation conditions. Thus, the samples prepared at pH = 2.5 from solution without saccharin (sample S1) contains 90 at.% Pt and 10 at.% Co, while the saccharin additions (sample S2) lead to the preparation of alloys having 88 at.% Pt and 12 at.% Co. When the electrochemical bath pH is increased to 5.5 (sample S3), alloys having 78 at.% Pt and 22 at.% Co is prepared, while the presence of saccharin (sample S4) leads to the preparation of catalysts with 15 at.% Pt and 85 at.% Co. The EDX results show that the CoPt alloy concentration is strongly dependent on the solution pH and composition. The lower percentage of Co in the samples prepared at the lower pH values (samples S1 and S2) is related to the fact that the metal hydroxides formation and absorption, as well as the dissolution of the freshly deposited metal atoms, influence the electrodeposition process and are highly correlated to the pH value of the electrochemical bath. The same evolution of the Co percent vs. bath pH value has been reported by Tian et al. [62] for electrodeposited CoNi thin films.

In their work, Tian et al. show that a lower pH value favors the dissolution of freshly deposited metal and depresses the metal hydroxide formation and absorption. Thus, the alloy composition can be tuned by changing the electrochemical bath characteristics (pH and saccharin). The addition of saccharin during electrodeposition leads to a higher Co content in the samples for both pH values. This behavior is attributed to the decreases of the H^+^ concentration at the electrodeposition surface when saccharin is added into the electrochemical bath at the same time as the increase of the concentration of the Co hydroxide species and, consequently, to the increase of Co ions available for the reduction to metal. The same behavior was observed earlier in the electrodeposition of CoPt at the controlled potential in quiescent solutions [31]. The voltammetry studies presented in our previous papers (see refs. [31,35]) show that the addition of saccharin to a CoPt solution changes the position and height of the peaks, leading to a decrease in the hydrogen partial current densities. This result shows that the concentration of the electroactive H^+^ species at the electrode surface is smaller when saccharin is added to the electrochemical bath, leading to the local increase of the pH value and to the preparation of CoPt deposits with higher Co content.

**Figure 4:**
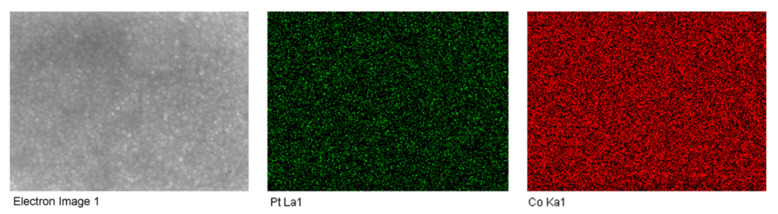
Elemental mapping of CoPt sample prepared at pH = 5 in the presence of saccharin.

The homogeneity of the samples’ composition has been determined by EDX (by performing analysis at different points of the surface sample, the results being presented in Tables S1–S4 in Supplementary Materials) and also by elemental mapping. Figure 4 presents the results of the elemental mapping of the sample prepared at pH = 5.5 in the presence of saccharin. It can be observed from Figure 4 the Pt and Co elements are uniformly distributed on the sample surface, showing a good sample homogeneity.

### 3.2. XRD Analysis of the CoPt Alloys

**Figure 5:**
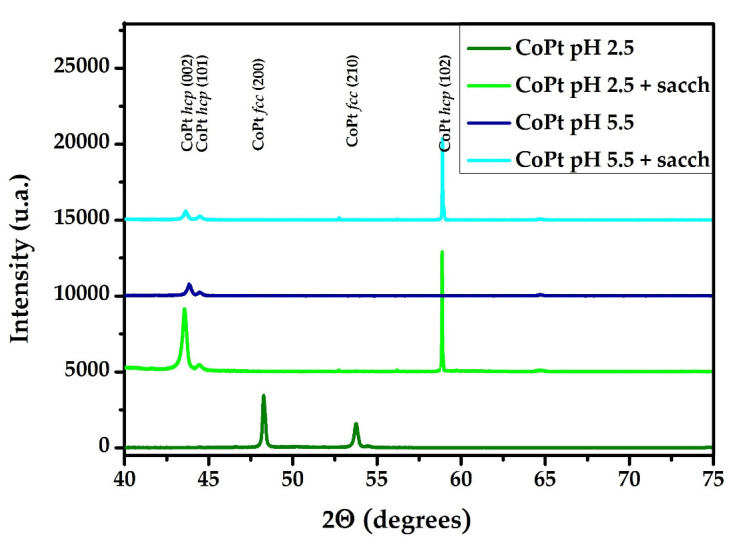
XRD patterns of CoPt alloys are prepared in different solutions.

The XRD data in Figure 5 show that the alloys prepared from the solution without additives at pH = 2.5 present fcc structure. In contrast, the addition of saccharin and the increase of the pH, lead to the formation of hcp structure. The XRD diffraction peaks at 2θ = 48.2 and 53.7 of the samples prepared at pH = 2.5 in the absence of saccharin can be assigned to the (200) and (210) fcc reflections, respectively. For the samples prepared at pH 5.5 or in the presence of saccharin, the diffraction peaks at 2θ = 48.2 and 53.7 disappear, and a new peak, characteristic of the hcp structure, appears at 2θ = 43.5, 44.5, 58.8. This reflection can be assigned to (002), (101) and (102) hcp structures as found by Cortes et al. [63]. The samples prepared at pH = 2.5 in the presence of saccharin also present the (002), (101) and (102) hcp reflections.

**Figure 6:**
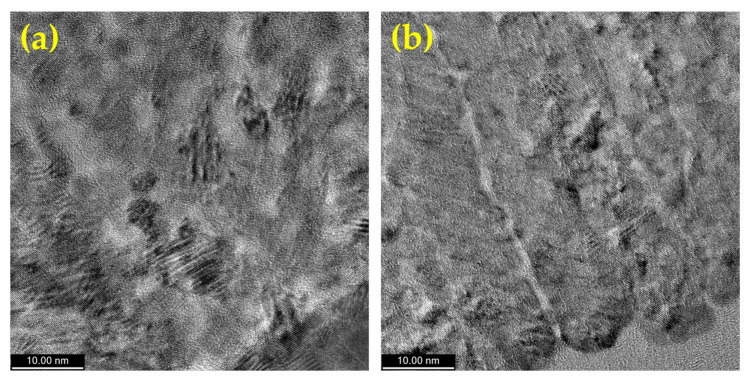
HR-TEM images of the cross-section of the electrodeposited CoPt alloy for various electrodeposition solution: (**a**) pH = 5.5 solutions without saccharin addition (sample S3), (**b**) pH = 5.5 solutions with saccharin addition (sample S4).

The average crystallite size of the as-prepared CoPt alloys was calculated based on the Scherrer equation, the results being well correlated with those obtained by using AFM measurement. The average crystallite size determined by using the Scherrer formula is only a few nanometers higher than the average grain size measured by the AFM, with the difference coming from the tip radius (during the measurements, tip radius of 2 nm was used). Our data clearly show that a single crystal forms each grain. The results (presented in Table 1) obtained by this method show that the crystallite size slightly decreases for the samples prepared at the same pH, when saccharin is added into the electrochemical bath. The increase of the pH value from 2.5 to 5.5 lead to an increase in the crystallite size from 54 nm (sample S1) to 88 nm (sample S3). This variation of the crystallite size is due to the increase of the Co content in the alloy. In order to clarify the alloy crystalline structure as a function of the synthesis conditions, we studied the cross-section of the CoPt thin films prepared at pH = 5.5 by means of HR-TEM; the results are presented in Figure 6. The HR-TEM images’ analysis shows that both samples are polycrystalline, presenting the hcp structure in accord with the XRD measurements. For both of the analyzed samples, the measured d-spacing of 2.10 Å and 2.01 Å, match the (002) and (101) Co-hcp orientation. A small number of amorphous compounds (presumably oxide/hydroxide) were also observed at the grains interface. These compounds are not visible on the XRD patterns due to the fact that they are present in a very small amount. Moreover, an important conclusion of the HR-TEM study is that when saccharin is added to the electrochemical bath, the obtained thin film exhibits columnar growth.

### 3.3. Reduction of 4-NP to 4-AP Using Synthesized Materials

**Figure 7:**
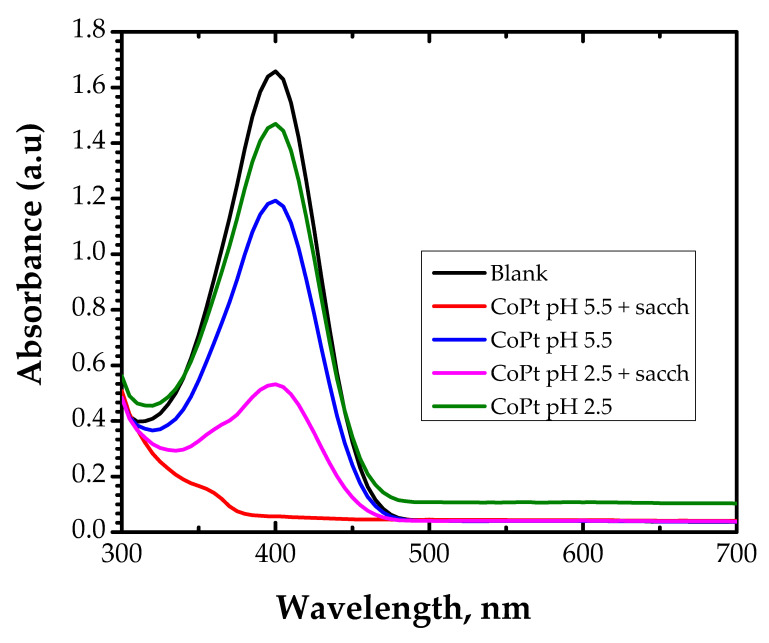
Absorption spectra of the conversion of 4-Nitrophanolate anion by the CoPt materials.

The 4-nitrophenol (4-NP) was reduced to 4-aminophenol (4-AP) in the presence of NaBH_4_ in order to establish the potential catalytic performance of all four synthesized materials, Figure 7.

From Figure 7, it can be observed that the catalytic performance of the investigated materials differs. The peak intensity at 400 nm did not decrease significantly even after 120 min of contact time of the CoPt samples prepared from an electrochemical bath without saccharin regardless of the electrochemical bath pH value (samples S1 and S3).

In the case of the CoPt sample prepared at pH = 2.5 in saccharin (sample S2), the absorbance at 400 nm decreased to approximately 0.5 after 120 min of contact time. The peak at 400 nm disappeared when catalyst CoPt prepared at pH = 5.5 in the presence of saccharin (sample S4) is used, showing the complete conversion of the nitro to the amino group.

Based on the obtained results, it can be concluded that the catalytic performance in the reduction of 4-NP to 4-AP (highlighted by UV-VIS measurements of the conversion of 4-Nitrophanolate anion to the 4-aminobenzoate anion) of the samples prepared with saccharin is higher than the catalytic performance of the samples prepared from the electrochemical bath without saccharin. This observation can be explained by the differences in the crystalline structure, crystallite size, and surface characteristics (such as surface roughness) of the catalyst.

Table 1 summarizes the data concerning the conversion degree of the nitro to amino compounds after 120 min of contact time for all four prepared samples. The conversion degree has been calculated by reporting each sample’s absorbance peak intensity to the blank sample’s peak intensity and considering the blank sample’s conversion degree as 0%.

From Table 1, it can be noted that the electrodeposition (ED) bath characteristics (pH value and addition of saccharin) strongly influence the catalytic properties of the samples. Thus, the sample prepared at pH = 2.5 in the absence of saccharin (sample S1) manifests the lowest conversion degree for the 4-nitrophenol transformation reaction, even though the sample prepared in this condition has the highest Pt content. When saccharin is added to the electrochemical bath (sample S2), the conversion degree of the 4-nitrophenol is increased from 11.4% to 28.0%, an increase of 2.45 times. This behavior is related to the decrease of the crystallites, the increase of the surface roughness (leading to the increase of the sample surface) size as well as the change of the crystalline structure from cfc (structure obtained in the absence of saccharin, sample S1) in hcp (structure obtained when saccharin is added to the electrochemical bath, sample S2). Further, the conversion degree of the 4-nitrophenol is increasing to 67.9% (representing an increase of 5.95 times, compared with the sample prepared at pH = 2.5 whiteout saccharin, when the bath pH increased to 5.5 (sample S3) and respectively to 96.5% (representing an increase of 8.46 time, compared with the sample prepared at pH = 2.5 whiteout saccharin) when saccharin is added into the electrochemical bath (sample S4), despite the decrease of the Pt content in the alloy. This behavior is strongly related to the crystalline structure of the alloy and the orientation of the crystallites as was previously demonstrated by our team [64]: in the case of the sample prepared at pH = 5.5 (electrochemical bath with and without saccharin), the variation in crystallite orientation results in an increase in Pt content nearer the sample surface, contributing to an increase in the sample’s catalytic efficiency. The increase of the surface roughness also contributes to the increase of the sample catalytic surface, leading to an increase in the conversion degree.

**Figure 8:**
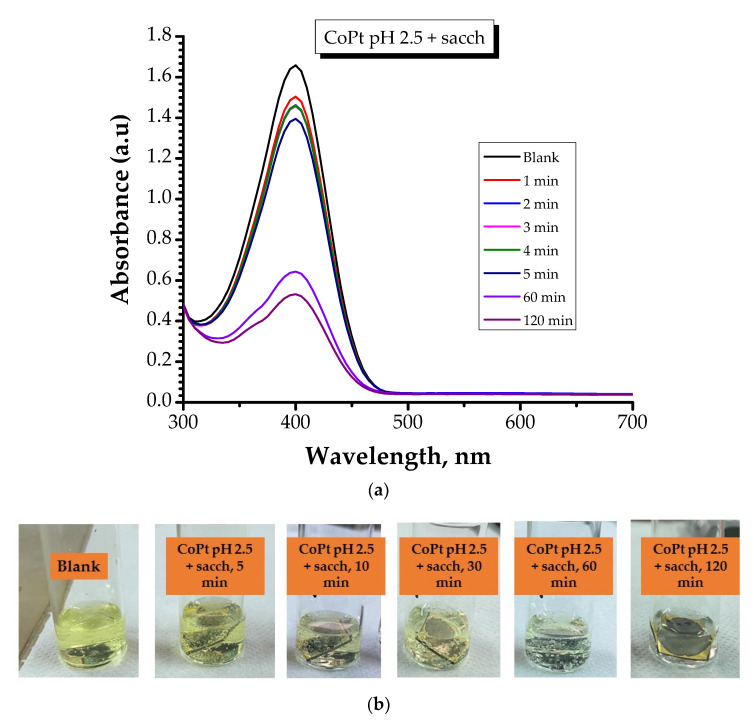

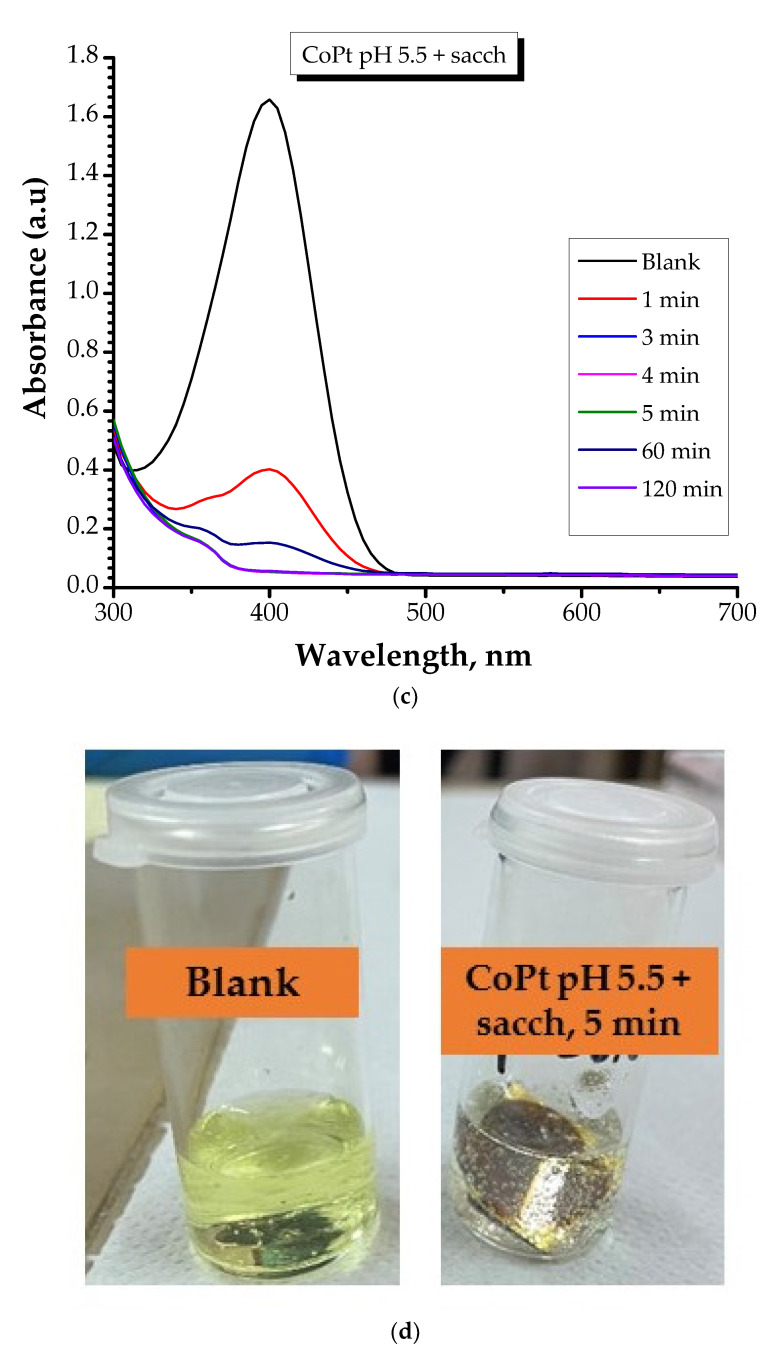
Absorption spectra of the conversion of 4-Nitrophanolate anion over time by: (**a**) CoPt pH 2.5 + sacch (sample S2) and (**c**) CoPt pH 5.5 + sacch samples measured for 120 min contact time; (sample S4); (**b**) Optical view of the color of the solution mixture for CoPt pH 2.5 + sacch (sample S2); (**d**) Optical view of the color of the solution mixture for CoPt pH 5.5 + sacch (sample 4).

Moreover, the effect of contact time was explored to more clearly explain the advantages of the CoPt alloy prepared at pH = 2.5 in the presence of saccharin and CoPt alloy prepared at pH = 5.5, also in the presence of saccharin samples. The results of the absorbance vs. time are illustrated in Figure 8.

After reviewing the data, it can be highlighted that: (i) in the case of CoPt prepared at pH = 2.5 in the presence of saccharin (sample S2), with increasing contact time, the color of the mixture progressively diminished; (ii) in the case of CoPt prepared at pH = 5.5 in the presence of saccharin (sample S4), the absorption peak at 400 nm\disappeared after approximately 5 min of contact time, indicating that the 4-NP had been reduced and this type of material can be considered effective for 4-NP reduction.

The findings of this study were compared to those reported in the literature in Table 2.

Ayodhya and Veerabhadram investigated the catalytic activity of g-C_3_N_4_/CuS composite to reduce 4-NP to 4-AP. The experiments were performed as follows: a volume of 40 μL of 4-NP solution, 1.26 × 10^−2^ mol/L, was added into 2.5 mL of double distilled water and then mixed with 0.5 mL of freshly prepared aqueous NaBH_4_ solution, 0.5 M. Then, the catalyst was added to the yellow-colored solution. Their results demonstrated that a reaction time of 50 min is sufficient for 4-NP reduction [65].

In the case of material synthesized by EL-Sheshtawy and co-workers, namely Ag/g-C_3_N_4_/V_2_O_5_, the contact time needed to reduce 4-NP to 4-AP is 60 min [66]. The study was carried out under the following conditions: 2.8 mL deionized water and 40 μL NP (0.01 M) were mixed. The solution was stirred for one hour of contact time, and then a volume of 80 μL freshly prepared NaBH_4_ solution (0.5 M) was added. In the last step, 10 μL of catalyst (5 mg/mL) was added to the mixed solution.

Saxena and Saxena demonstrated that the performance of ACMNPs materials differs in function of working parameters. The authors performed the work by adding 0.2 mL of 10 mM NaBH_4_ to 5 mL of 0.1 mM 4-NP solution. For example, as the catalyst amount increased from 10 to 15 mg, the catalytic reduction was achieved in 18 and 14 min. On the other hand, a reaction time of 9 and 6 min is necessary if a catalyst amount of 20 and 25 mg is used, respectively. The authors showed that the temperature has an impact on the catalytic reduction as well: when an amount of 20 mg of catalyst is used, and the temperature is raised from 25 to 55 °C, the rate of catalysis increases while the time required for catalysis decreases from 9 to 4 min [67].

The CoFe_2_O_4_/ZrMCM-41, 25% nanocomposite, shows a good catalytic activity (100%) for the reduction of 4-NP (6 min) using the following conditions: 2 mL of a 4-NP aqueous solution having a concentration of 0.2 mM were mixed with 0.5 mL of fresh NaBH_4_ solution, 20 mM [68].

The catalytic reduction of 4-AP using the material proposed by Yang and co-workers promises (24 min of reaction time) [69]. The catalytic activity of Ni-HPF was established based on the following work plan: 50 mg of Ni-HPF were mixed with an aqueous solution containing 5 mL of 4-NP, 0.1 mM and 0.5 mL of NaBH_4_, 0.5 M.

Recently, Kalantary and co-workers published a study that demonstrated that when Cu NPs-Fe_3_O_4_-SAlg is used, the reduction time of 4-NP is achieved in approximately 4 min. Also, they compared their results with the available literature [70]. The experiments were performed using 4-NP solution of 2.5 × 10^−3^ mol/L, and 2.5 × 10^−3^ mol/L of NaBH_4_. The amount of catalyst used was 10 mg.

The Fe3O_4_/CS-Me@Pd microcapsules show catalytic ability in reducing this type of pollutant (150–300 s) [71]. The reduction reaction was carried out with NaBH_4_ at room temperature. The freshly prepared NaBH_4_ solution (1 mg/mL) was added to 5 mL of 4-NP solution, 1 mg/mL, and the reaction started with the addition of the catalyst (6.0, 8.0, 10.0, 12.0 mg).

CoPt, rGO/CoPt, and rGO/CoPt/Ag catalysts showed a reduction time of 8, 4, and 1 min [15]. A volume of 0.1 mL of 4-NP, 0.005 mol/L and 2 mL of deionized water was added to the quartz cuvette. An amount of 1 mL of NaBH_4_ (0.2 mol/L) was also added. The reaction started after adding 60 μL of catalyst aqueous dispersion (2 mg/mL).

Co_25_Pt_75_, Co_50_Pt_50_ and Co_75_Pt_25_ alloy nanoparticles exhibited a catalytic performance for the reduction of 4-NP to 4-AP: 3 min, 4 min, and 2 min, respectively [32]. The authors proposed the following working conditions: 1 mL NaBH_4_, 0.2 M/L solution and 2 mL 4-NP, 0.005 M/L solution were taken to 100 μL Co_25_Pt_75_, Co_50_Pt_50_ and Co_75_Pt_25_ alloy NPs aqueous dispersion (2 mg, 2 mg/mL) was introduced.

GO, and Pd/rGO-H materials presented a reaction time of 60 min, while for Pd/GO-P material, a reaction time of 2 min was found to be sufficient [72]. The test was run in this manner: 4-NP solution (0.145 mM, 2.5 mL) was added to the cuvette, and 50 μL of the as-synthesized catalyst was added. Finally, 50 μL freshly prepared sodium borohydride solution, 1 M, was added.

The Bi_2_Te_3_−MoS_2_ layered heterostructures displayed a good catalytic performance (35 min) [73]. An amount of 10 mg of the catalyst was stirred into 100 mL of water to obtain dispersion. After that, 256 mg of 4-NP (18.4 mmol) was dissolved into the aqueous dispersion of the catalyst, followed by adding 1.51 g of NaBH_4_ (400 mmol) with constant stirring.

From Table 2, it can be observed that CoPt prepared at pH = 5.5 in the presence of saccharine shows a good catalytic activity, and it can be recommended as an alternative material in 4-NP reduction.

## 4. Conclusions

CoPt thin films with a thickness of 50 nm were prepared by electrodeposition by applying a potential of −0.8V/SCE for 2.5 s followed by −0.1 V/SCE for 1 s. The synthesis was carried out from stable hexachloroplatinate solutions at two different pH values (2.5 and 5.5, respectively), with or without adding saccharin as an organic additive. The catalytic properties of the as-prepared samples were tested for the conversion of the 4-Nitrophenol into the 4-Aminophenol. The microstructures of the samples were analyzed by different techniques (SEM, TEM and AFM). The measurements showed that the sample surface is completely covered with a continuous CoPt alloy thin film. EDX performed the compositional analysis of the samples, showing that the Pt concentration of the alloy thin films can be tuned from 15 to 90%, strongly dependent on the electrochemical bath characteristics. Thus, increasing the pH of the electrodeposition coating solution from 2.5 to 5.5 decreases the Pt content in the electrodeposited alloy. The addition of saccharin results in higher Co content and, respectively, lower Pt content in CoPt films produced by this method, regardless of the electrodeposition bath pH value. Saccharin also has important effects on the microstructure and crystal structure of the prepared CoPt thin films, thus influencing the grain size and the surface roughness. Consequently, these different properties of the samples strongly influence the catalytic properties of the thin films for the reduction of the 4-NP.

The order of reaction completion followed the order: CoPt pH = 5.5 + sacch > CoPt pH = 2.5 + sacch > CoPt pH = 5.5 > CoPt pH = 2.5. This fact highlighted that the CoPt prepared at pH = 5.5 in the presence of the saccharin sample is the most promising catalyst. This good catalytic performance can be attributed to its crystalline structure.

This study will be extended by investigating other parameters (such as the applied potential or solution pH) that influence the physical properties of the CoPt alloy and that impact the catalytic properties of the CoPt thin film.

## Figures and Tables

**Table 1:** Physical and chemical parameters of the CoPt catalysts.

Sample	Bath pH	Saccharin Addition	ED Time (s)	Surface Roughness (nm)	Crystalline Structure	Crystallite Size (nm)		Alloy Composition		Conversion Degree after 120 min, %
						XRD	AFM	% Pt	% Co	
S1	2.5	NO	25	7.5	cfc	54	55	90	10	11.4
S2		YES	30	9	hcp	46	50	88	12	28.0
S3	5.5	NO	30	9.2	hcp	88	90	78	22	67.9
S4		YES	35	10.6	hcp	74	75	15	85	96.5

**Table 2:** Comparison of the catalytic activity with available literature for catalytic reduction of 4-NP.

Material	Reaction Time for the Reduction of 4-NP	References
g-C_3_N_4_/CuS composite	50 min	[65]
Ag/g-C_3_N_4_/V_2_O_5_	60 min	[66]
ACMNP (10 mg)	18 min	[67]
ACMNP (15 mg)	14 min	[67]
ACMNP (20 mg)	9 min	[67]
ACMNP (25 mg)	6 min	[67]
CoFe_2_O_4_/ZrMCM-41 25% nanocomposite	6 min	[68]
Nickel-coated hyperporous polymer foam (Ni-HPF)	24 min	[69]
Cu NPs-Fe_3_O4-SAlg	~4 min	[70]
Fe_3_O_4_/CS-Me@Pd microcapsules	~2.5–5 min	[71]
CoPt, rGO/CoPt, and rGO/CoPt/Ag catalysts	8, 4, 1 min	[15]
Co_25_Pt_75_, Co_50_Pt_50_ and Co_75_Pt_25_ alloy nanoparticles	3, 4, and 2 min	[32]
GO	60 min	[72]
Pd/rGO-H	2 min	[72]
Bi_2_Te_3_−MoS_2_ layered heterostructures	35 min	[73]
CoPt pH = 5.5 + sacch	~5 min	Present study

## Data Availability

The data presented in this study are available on request from the corresponding author.

## Supplementary Materials

The following supporting information can be downloaded at: https://www.mdpi.com/article/10.3390/ma15186250/s1, Figure S1: Lines profile of the electrodeposited CoPt alloy for various electrodeposition solutions; Figure S2: AFM images of the Au support tougetter with the corresponding surface statistics; Figure S3: EDX spectra of the electrodeposited CoPt alloy for various electrodeposition solution; Table S1: EDX analysis of the electrodeposited CoPt thin film from electrochemical bath at pH = 2.5, in absence of saccharin; Table S2: EDX analysis of the electrodeposited CoPt thin film from electrochemical bath at pH = 2.5, in presence of saccharin; Table S3: EDX analysis of the electrodeposited CoPt thin film from electrochemical bath at pH = 5.5, in absence of saccharin; Table S4: EDX analysis of the electrodeposited CoPt thin film from electrochemical bath at pH = 5.5, in presence of saccharin.
