# Supplementary material for: Effect of the Preparation Conditions on the Catalytic Properties of CoPt for Highly Efficient 4-Nitrophenol Reduction
Source: Materials (Basel). 2022 Sep 8;15(18):6250. doi: 10.3390/ma15186250 (PMC9501049; doi:10.3390/ma15186250)
Supplement: Supplementary file 1 [file materials-15-06250-s001.zip › materials-1805722-supplementary.pdf]

Effect of the Preparation Conditions on the Catalytic Properties of CoPt for Highly Efficient 4-Nitrophenol Reduction

Oana-Georgiana Dragos-Pinzaru [1], Gabriela Buema [1,*], Daniel Gherca [1,*], Ibro Tabakovic [2] and Nicoleta Lupu [1]

[1] National Institute of R&D for Technical Physics, 700050 Iasi, Romania
[2] ECE Department, University of Minnesota, Minneapolis, MN 55435, USA
* Correspondence: gbuema@phys-iasi.ro (G.B.); dgherca@phys-iasi.ro (D.G.)

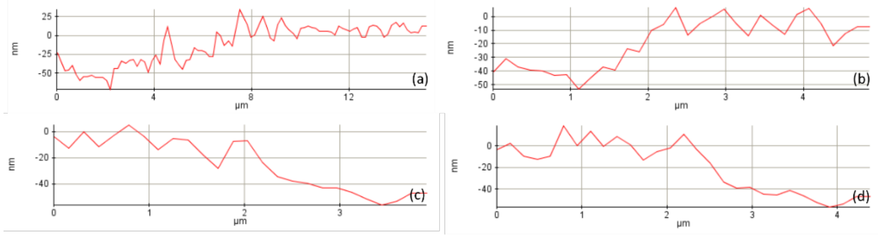

Figure S1. Lines profile of the electrodeposited CoPt alloy for various electrodeposition solutions: (a) pH=2.5 solutions without saccharine addition, (b) pH=2.5 solutions with saccharine addition, (c) pH=5.5 solutions without saccharine addition and (d) pH=5.5 solutions with saccharine addition

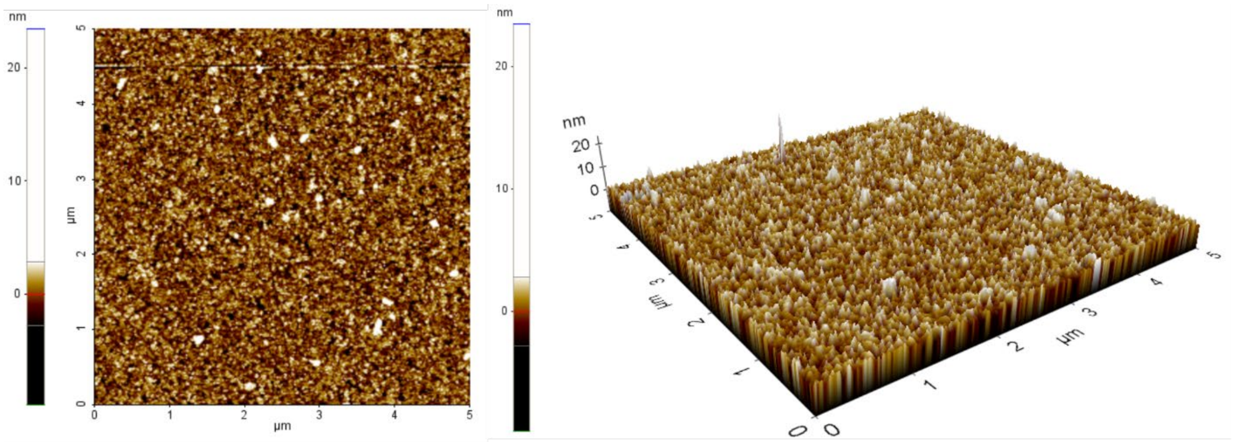

Statistics

Region	Min(nm)	Max(nm)	Mid(nm)	Mean(nm)	Rpv(nm)	Rq(nm)	Ra(nm)
Red	-8.045	8.455	0.205	-0.004	16.501	1.419	1.099

Figure S2. AFM images of the Au support tougetter with the corresponding surface statistics

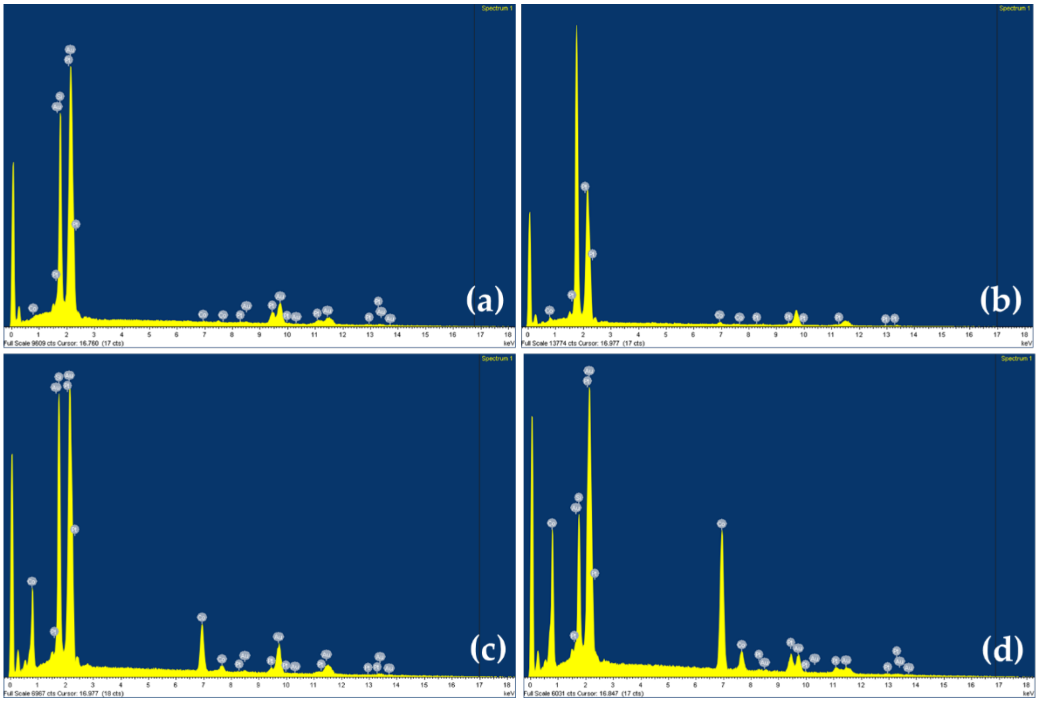

Figure S3. EDX spectra of the electrodeposited CoPt alloy for various electrodeposition solution: (a) pH=2.5 solutions without saccharine addition, (b) pH=2.5 solutions with saccharine addition, (c) pH=5.5 solutions without saccharine addition and (d) pH=5.5 solutions with saccharine addition

Table S1: EDX analysis of the electrodeposited CoPt thin film from electrochemical bath at pH = 2.5, in absence of saccharin

No. crt.	ED bath pH	Addition of saccharin	Alloy composition	
			% Pt	%Co
1	2.5	NO	90	10
2			92	8
3			90.5	9.5
4			88	12

Table S2: EDX analysis of the electrodeposited CoPt thin film from electrochemical bath at pH = 2.5, in presence of saccharin

No. crt.	ED bath pH	Addition of saccharin	Alloy composition	
			% Pt	%Co
1	2.5	YES	88	12
2			89	11
3			88.5	11.5
4			90	10

Table S3: EDX analysis of the electrodeposited CoPt thin film from electrochemical bath at pH = 5.5, in absence of saccharin

No. crt.	ED bath pH	Addition of saccharin	Alloy composition	
			% Pt	%Co
1	5.5	NO	78	22
2			78.5	21.5
3			80	20
4			77	23

Table S4: EDX analysis of the electrodeposited CoPt thin film from electrochemical bath at pH = 5.5, in presence of saccharin

No. crt.	ED bath pH	Addition of saccharin	Alloy composition	
			% Pt	%Co
1	5.5	YES	15	85
2			16	84
3			15.5	84.5
4			14.5	85.5
